# Olanzapine-Induced Therapeutic Hypothermia Attenuates Renal Injury in Rats after Asphyxial Cardiac Arrest and Resuscitation

**DOI:** 10.3390/antiox11030443

**Published:** 2022-02-23

**Authors:** Tsendsuren Tungalag, Yeo-Jin Yoo, Hyun-Jin Tae, Dong Kwon Yang

**Affiliations:** 1 Department of Veterinary Pharmacology and Toxicology, College of Veterinary Medicine, Jeonbuk National University, Iksan 54596, Jeollabuk-do, Korea; mgljuuh@jbnu.ac.kr; 2 Department of Veterinary Anatomy and Toxicology, College of Veterinary Medicine and Bio-Safety Research Institute, Jeonbuk National University, Iksan 54596, Jeollabuk-do, Korea; yooyeos@jbnu.ac.kr

**Keywords:** cardiac arrest, ROSC, ischemia–reperfusion injury, oxidative stress, reactive oxygen species, Sirt3

## Abstract

Return of spontaneous circulation (ROSC) through cardiopulmonary resuscitation (CPR) after cardiac arrest (CA) causes post-cardiac arrest syndrome (PCAS) due to dysfunction in various organs, which provokes acute kidney injury because of renal ischemia-reperfusion injury. Therapeutic hypothermia (TH) can reduce PCAS after CA and ROSC. However, it needs to be more sophisticated and effective. Hence, we aimed to elucidate the protective effects of olanzapine-induced TH against renal injury in asphyxial CA-induced rats. Every rat’s body temperature was maintained at 33 °C for 6 h after administering olanzapine post-CA and ROSC. Olanzapine-induced TH dramatically increased the survival rate of the rats and ameliorated renal tissue damage. Moreover, it suppressed oxidative stress responses through preservation of mitochondrial function and endoplasmic reticulum stress as the main contributor of oxidative stress. Notably, these actions of olanzapine-induced TH were mediated through the Sirt3-related signaling pathway, including the maintenance of Sirt3 and FOXO3a protein expression and the activation of AMPKα and superoxide dismutase 1 (SOD2, a mitochondrial antioxidant). This study is the first to disclose the protective effects of olanzapine-induced TH against renal injury after CA and ROSC, suggesting that olanzapine-induced TH could be utilized for treating CA followed by ROSC.

## 1. Introduction

Cardiac arrest (CA) is the interruption of blood flow because of sudden heart beating that further causes ischemia, neurological injury, and unconsciousness [1]. CA can occur either inside a hospital or outside a hospital. The incidence of CA was 10.2 individuals per 1000 hospital admissions in 2019, as per the 2019 Get With The Guidelines data; and the incidence of CA in patients outside hospitals was 76.5 individuals per 100,000 in America [2]. The survival rates of both are ultimately poor, being less than 22% and 15%, respectively [3,4]. 

Unfortunately, individuals who survive CA as a result of cardiopulmonary resuscitation (CPR) have severe medical problems, including impaired consciousness and cognitive deficits [5]. Furthermore, multiple organ dysfunction commonly arises after return of spontaneous circulation (ROSC), contributing to a high mortality rate after surviving from CA [5]. The brain tissue is considered to be the organ most effected by CA; therefore, brain injury after ROSC has been intensively studied [6,7]. In fact, dysfunctions in various organs commonly occur after CA and ROSC. Some examples are cardiogenic shock, respiratory dysfunction, liver failure, and acute renal failure [8]. 

In a recent study, acute kidney injury (AKI) occurred in 69.8% of CA survivors. Furthermore, the death rate was 69.4% of AKI patients in comparison with 52.0% of non-AKI patients, indicating that AKI is closely associated with the high mortality of CA, although it receives less attention than brain injury after CA [9]. AKI after CPR is closely related to ischemia–reperfusion (IR) injury [10]. Renal IR injury generates oxygen radical species, including superoxide anions, radical hydroxyls, and hydrogen peroxide, thereby further causing renal damage through oxidative stress [11]. Therefore, given the high mortality of patients resuscitated from CA after successful ROSC, additional therapeutic strategies are needed to attenuate multiple organ dysfunction, particularly renal injury. 

Since it was first reported in 1943, therapeutic hypothermia (TH) has long been used for patients who receive ROSC after CA in order to reduce mortality and prevent neurological disorders [12]. There are several clinically applied cooling methods for the induction of TH, including conventional, surface, and intravascular cooling methods [13]. The conventional cooling method uses cold saline infusion and ice packs. The surface cooling method involves the use of blankets or gel pads filled with circulating cold fluid for wrapping around the patients. Finally, the intravascular cooling method uses intravascular catheters that are placed into central veins to circulate temperature-controlled cold saline. This method is considered the most reliable one. However, all these methods have serious limitations: e.g., they are labor intensive, are not effective at maintaining the target temperature, and are associated with risks of skin burns and irritation. Therefore, a more sophisticated and effective method to induce TH is required. Here, we aimed to establish the protective effects of TH induced by olanzapine, an antipsychotic drug, against renal injury following CA. Indeed, TH, which lowers the body temperature to 32–35 °C, has been demonstrated the increase the survival rate and attenuate the symptoms of many complications, particularly neurological disorders [14,15]. Many studies have described the beneficial effects of TH, including reduced oxygen demand in ischemic regions, metabolic acidosis, and an inflammatory response [16,17]. Serum analysis in CA patients after ROSC has also revealed that TH has preventive effects against inflammation [18] and oxidative damage [19]. TH was found to inhibit the apoptotic process in the brain tissue after CA and ROSC in pig model [20]. Conversely, other studies have shown that TH is not beneficial to CA patients after ROSC [21,22,23]. Since the efficacy of MTH remains controversial, the protective mechanisms of MTH on CA patients need to be further elucidated. 

In this study, we used the olanzapine possessing the hypothermic effect as an antipychotic drug to develop the sophisticated and effective technique for inducing the TH. We demonstrated that therapeutic hypothermia (TH) induced by olanzapine exerts a preventive effect against renal IR injury after CA and ROSC. It ameliorates oxidative stress by suppressing the mitochondrial dysfunction and the endoplasmic reticulum (ER) stress.

## 2. Materials and Methods

### 2.1. Animal Studies 

Male Sprague–Dawley rats weighing 280–310 g obtained from the Experimental Animal Center of Jeonbuk National University (Jeonju, South Korea) were individually housed under controlled environment with the temperature (23 ± 2 °C) and humidity (60 ± 10%) control with a 12-h light/dark cycle. Food and water were available ad libitum. The rats were randomly assigned to three groups: sham (*n* = 6), CA + normothermia (NT, *n* = 27), and CA + hypothermia (HT, *n* = 17) groups. All the procedures were approved by the Institutional Animal Care and Use Committee of Jeonbuk National University (JBNU 2020-084).

### 2.2. CA and CPR Procedures

**Figure 1:**
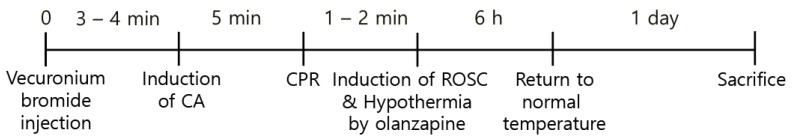
Experimental design for the induction of CA, ROSC, and olanzapine-induced TH.

The rodent ventilator (Harvard Apparatus, Holliston, MA, USA) and 2–3% isoflu-rane were used for anesthesia and ventilation during the CA and CPR procedures. A pulse oximeter connected to the left leg was used for monitoring the peripheral oxygen saturation (SpO_2_). Furthermore, electrocardiography (ECG) readings and the mean arterial pressure (MAP) were constantly monitored through the limbs and left femoral artery, respectively. After 5 min, 2 mg/kg vecuronium bromide was intravenously injected for the induction of CA [24] followed by ceasing of the ventilation. The induction of CA was considered when MAP fell below 20 mmHg [25], resulting in pulseless electrical activity (PEA), which took 3–4 min after the injection of the drug. After 5 min following the induction of CA, a 0.005 mg/kg bolus of epinephrine and 1 mg/kg sodium bicarbonate were intravenously administered. Subsequently, CPR was performed by mechanical chest compression on the chest of the rats at a depth of one-third of the anteroposterior region, at a rate of 300/min, and with 100% oxygen supply using an animal CPR machine (Jeung Do Bio & Plant Co., Ltd., Seoul, South Korea) until MAP returned to 60 mmHg [26]. During the CPR procedure, ECG and body temperature were constantly monitored (Figure 1).

### 2.3. Induction of Hypothermia

For the induction of hypothermia, olanzapine (10 mg/kg) was immediately injected intraperitoneally after the induction of ROSC to reduce the body temperature to 33 °C. At 6 h after the body temperature was dropped, the body temperature was raised to 37 °C as a normal temperature via heating pad. The rectal temperature was measured using a rectal thermometer (Homeothermic Monitoring System; Harvard Apparatus, Holliston, MA, USA). The anesthesia with isoflurane was maintained to prevent hypothermic shock during the experiment. Then, 24 h after CPR was performed, all the rats were sacrificed for further analyses. 

### 2.4. Hematoxylin and Eosin (H&E) and Periodic Acid–Schiff (PAS) Staining

The kidney tissues were perfused with 0.1 M phosphate-buffered saline (PBS) and fixed with 4% paraformaldehyde. They were further processed to embed them with paraffin and dissected into 7 µm thick slices. For the H&E staining, the sections were dehydrated with ethanol, stained with H&E, and mounted with Canada balsam (Kanto Chemical, Tokyo, Japan). For PAS staining, the PAS Stain Kit (Azer Scientific Inc., Morgantown, PA, USA) was used according to the manufacturer’s instructions. Histological glomerular injury was scored as follows according to the damaged regions by referring to previously established procedures [27]: 0: normal; 1: less than 25%; 2: between 25% and 50%; 3: between 50% and 75%; and 4: between 75% and 100%.

### 2.5. Measurement of Serum Urea Nitrogen (BUN) and Creatinine

The serum was obtained from the blood in inferior vena cava by centrifugation at 4000× *g* for 15 min. Serum BUN and creatinine levels were determined by Olympus AU 2700 Analyzer (Olympus Optical Co., Ltd., Tokyo, Japan).

### 2.6. Immunostaining for Terminal Deoxynucleotidyl Transferase dUTP End Labeling (TUNEL); Intracellular Reactive Oxygen Species (ROS) Production; Mitochondrial Membrane Potential (MMP); Sirt3 and FOXO3a Proteins

For immunostaining assays, the kidney tissues were cryopreserved with OCT compound (Sakura Finetek, Torrance, CA, USA) and dissected to 5 µm thickness. They were then fixed with 4% paraformaldehyde for 2 min at room temperature (RT) followed by washing thrice with PBS. The measurement of apoptotic cell death was performed by TUNEL staining using the commercially available Kit (Roche Diagnostics, Manheim, Germany). In brief, they were permeabilized with 0.3% Triton-X 100 dissolved with 0.1% sodium citrate for 2 min on 4 °C, and incubated at 37 °C for 1 h. For measurement of ROS production, the tissues were incubated with 3 µM DCFH-DA dye (ThermoFisher Scientific Inc., Waltham, MA, USA) for 20 min at RT [28]. MMP was measured by incubating with 5 μg/mL JC-1 dye (ThermoFisher Scientific Inc.) for 15 min at 37 °C, then observed using a fluorescence microscope (Oxford Instruments, Oxfordshire, UK). Fluorescence intensity was assessed with green fluorescence (550 nm excitation/600 nm emission) and red fluorescence (485 nm excitation/535 nm emission). For the immunostaining of Sirt3 and FOXO3a proteins, sectioned tissues were blocked with 20% donkey serum and 5% BSA in 0.1% TBST for 1 h and further incubated with Sirt3 (Cell Signaling) or FOXO3a (Cell Signaling) antibodies for 1 h at RT. Finally, Alexa488-conjugated secondary antibody (Jackson ImmunoResearch Lab) was treated after washing thrice with PBS.

### 2.7. Western Blot Analyses

The kidney protein extracts were obtained with RIPA buffer (LPS Solution, Daejeon, Korea) containing protease inhibitors (ThermoFisher Scientific Inc.) and phosphatase inhibitors (Roche Diagnostics). After being separated and transferred to PVDF membranes, they were blocked with 5% BSA in 0.1% TBST for 1 h at RT. The membranes were sequentially incubated with primary antibodies overnight at 4 °C and secondary antibodies (Jackson ImmunoResearch Lab., Inc., WestGrove, PA, USA) for 1 h at RT. Protein bands were detected and quantified using the Immobilon Western Chemiluminescence Kit (Millipore Corp., Billerica, MA. USA) and the UVITEC Mini HD9 System equipped with HD imaging software (Cleaver Scientific Ltd., Warwickshire, UK). The primary antibodies used in the present study were as follows: B-cell lymphoma 2 (Bcl-2; 1:1000, Santa Cruz Biotechnology, TX, USA), Bcl-2-associated X protein (Bax; 1:1000, Cell Signaling, Beverly, CA, USA), pro-caspase 3 (1:1000, Cell Signaling), cleaved caspase 3 (1:1000, Cell Signaling), superoxide dismutase 1 (SOD1; 1:1000, Santa Cruz), catalase (1:1000, Cell Signaling), glutathione peroxidase (GPx; 1:1000, Cell Signaling), SOD2 (1:1000, Cell Signaling), acetylated SOD2 (Ac-SOD2; Abcam, Cambridge, UK), mitochondrial complex II protein (1:1000, Abcam), pancreatic ER kinase (PERK; 1:1000, Cell Signaling), phosphorylated PERK (p-PERK; 1:1000, Cell Signaling), eukaryotic translation initiation factor 2 (eIF2; 1:1000, Cell Signaling), phosphorylated eIF2 (p-eIF2; 1:1000, Cell Signaling), activating transcription factor 4 (ATF4; 1:1000, Cell Signaling), C/EBP homologous protein (CHOP; 1:1000, Cell Signaling), growth arrest and DNA damage-inducible protein (GADD45; 1:1000, Cell Signaling), inositol-requiring enzyme (IRE1; 1:500, Cell Signaling), AMP-activated protein kinase (AMPK; 1:500, Cell Signaling), phosphorylated AMPK (p-AMPK; 1:500, Cell Signaling), and forkhead box O3 (FOXO3a; 1:500, Cell Signaling) antibodies.

### 2.8. Quantitative Real-Time Polymerase Chain Reaction (qRT-PCR)

Total RNA was isolated by commercially available kit (New England Biolabs Inc., Ipswich, MA, USA). The reverse transcription with 1 μg of total RNA was performed by the GoScript Reverse Transcription System Kit (Promega Co., Madison, WI, USA). qRT-PCR was performed using the TaKara Thermal Cycler Dice Real Time System (Takara Bio. Inc., Shiga, Japan) with the TOPreal™ qPCR 2X PreMIX Kit (Enzynomics Co., Daejeon, Korea). The primer sequences are shown in Table 1.

### 2.9. Statistical Analyses

Kaplan–Meier analysis with the log-rank was used for the survival rate. One-way analysis of variance (ANOVA) with a Bonferroni post hoc test was used for statistical analyses by Prism 8.02 (GraphPad Software Inc., San Diego, CA, USA). Data are expressed as mean ± SEM. Significant was considered at *p*-values < 0.05.

## 3. Results

### 3.1. Olanzapine-Induced TH Improves Overall Survival in CA-Induced Rats

**Figure 2:**
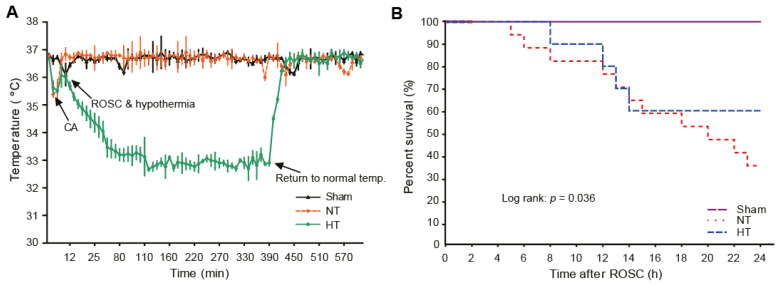
Olanzapine-induced TH improves overall survival in CA-induced rats. (**A**) Body temperature changes after the induction of CA. (**B**) The survival rate in rats after CA and ROSC with or without olanzapine-induced TH based on Kaplan–Meier analysis. TH, therapeutic hypothermia; CA, cardiac arrest; ROSC, return of spontaneous circulation; NT, normothermia; HT, hypothermia.

To induce hypothermia in CA-induced rats, olanzapine was intraperitoneally injected at a concentration of 10 mg/kg immediately after ROSC. As shown in Figure 2A, it took less than 5 min for body temperature to fall to 33 °C after olanzapine injection. After 6 h, when hypothermia was induced, the rat was re-warmed to normal body temperature via heating pad (Figure 2A). In addition, the survival rate up to one day after ROSC revealed that the survival rate in the CA + normothermia group was significantly reduced. It dramatically increased in the CA + hypothermia group (Figure 2B). 

### 3.2. Olanzapine-Induced TH Attenuates Renal Injury in CA-Induced Rats

**Figure 3:**
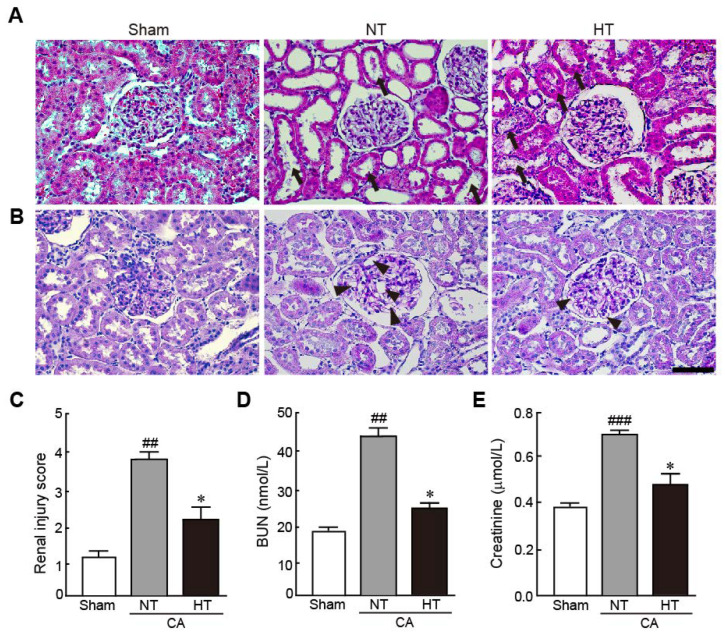
Olanzapine-induced TH attenuates renal injury in CA-induced rats. Representative photographs of H&E (**A**) and PAS staining (**B**) using renal tissues from rats after CA and ROSC with or without olanzapine-induced TH (*n* = 5 per group). Arrows and arrowheads indicate brush borders in renal tubules and glomerular capillaries. (**C**) The renal injury scores of rats after CA and ROSC with or without olanzapine-induced TH. Serum BUN (**D**) and creatine (**E**) levels were measured in rat tissues after CA and ROSC with or without olanzapine-induced TH (*n* = 5 per group). Significance is represented as ## *p* < 0.01 or ### *p* < 0.001 vs. the sham group; * *p* < 0.05 vs. the CA + NT group. CA, cardiac arrest; TH, therapeutic hypothermia; NT, normothermia; HT, hypothermia. Scale bar, 50 µm.

To elucidate the preventive effect of olanzapine-induced TH against renal injury in CA-induced rats, the pathohistological changes in renal tissues and serum levels of BUN and creatinine, which are renal injury markers, were evaluated. The renal tubules were severely dilated and brush borders were lost with necrosis in the CA + normothermia group (Figure 3A). In addition, the glomerular capillaries were severely dilated, as revealed by PAS staining (Figure 3B). However, these pathological changes were attenuated in the CA + hypothermia group. Therefore, due to the percentages of damaged regions in the renal tissue, the renal injury scores significantly decreased in the CA + hypothermia group; however, the scores dramatically increased in the CA + normothermia group (2.3 and 3.8, respectively) (Figure 3C). Similarly, serum levels of BUN and creatinine, which are renal injury indicators, dramatically increased in the CA + normothermia group (2.3- and 2.2-fold increases in BUN and creatinine levels in the CA + normothermia group, respectively, vs. sham group). However, these increased levels were significantly attenuated when hypothermia was applied after inducing CA in comparison with the levels in the sham group (1.2- and 1.3-fold increases in BUN and creatinine levels in the CA + hypothermia group, respectively, vs. the sham group). These results indicate that olanzapine-induced TH can effectively protect renal tissues from CA.

### 3.3. Olanzapine-Induced TH Inhibits CA-Induced Apoptotic Cell Death in Renal Tissues

**Figure 4:**
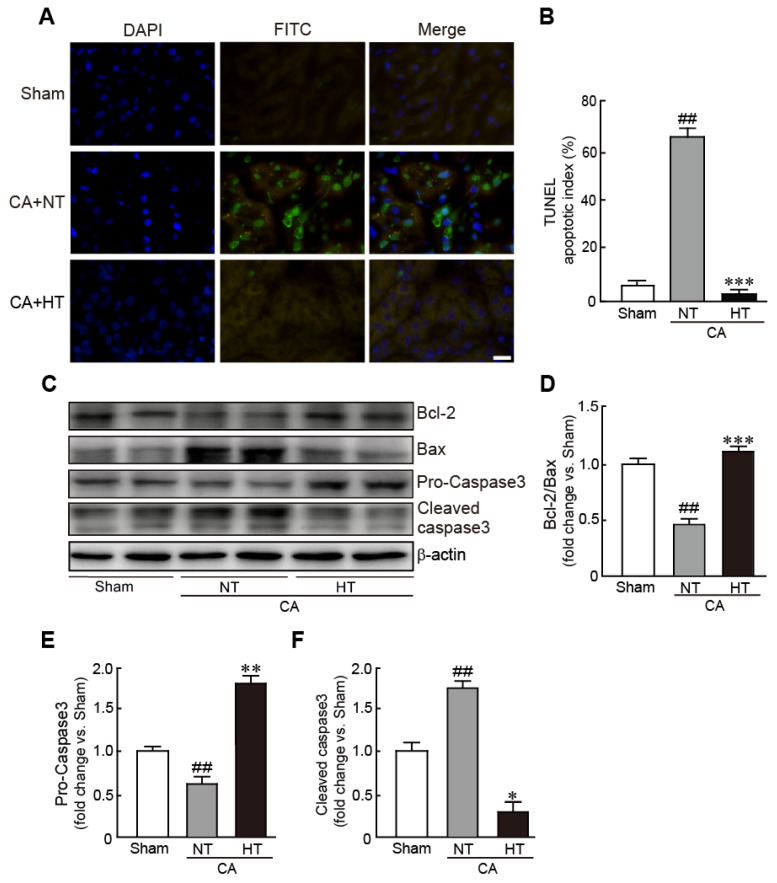
Olanzapine-induced TH inhibits CA-induced apoptotic cell death in renal tissues. (**A**) Representative photographs of TUNEL staining of rat tissues after CA and ROSC with or without olanzapine-induced TH. The apoptotic index (**B**) was determined by calculating the percentages of TUNEL-positive and total cells. (**C**–**F**) Western blot analysis for the expression of Bcl-2, Bax, pro-caspase 3, cleaved caspase 3, and *β*-actin proteins in renal tissues after CA and ROSC with or without olanzapine-induced TH (*n* = 3 per group). Significance is represented as ## *p* < 0.01 vs. the sham group; * *p* < 0.05, ** *p* < 0.01, and *** *p* < 0.001 vs. the CA + NT group. CA, cardiac arrest; TH, therapeutic hypothermia; NT, normothermia; HT, hypothermia. Scale bar, 100 µm.

For determining whether hypothermia inhibits apoptotic cell death in CA-induced rats, the apoptotic cells were observed with a TUNEL assay. The renal tissues from the CA + normothermia group had more apoptotic cells than those from the sham group. Apoptosis in the CA + hypothermia group was dramatically attenuated, with the portion of apoptotic cells being similar to that in the sham group (59.3% and 3.7% in the CA + normothermia and CA + hypothermia groups, respectively) (Figure 4A,B). Western blot analysis also revealed a significant decrease in Bcl-2 expression (apoptosis inhibitory protein) and a significant in Bax expression (apoptosis-induced protein) in the CA + normothermia group (0.4-fold decrease in Bcl-2/Bax levels in the CA + hypothermia group vs. the sham group). However, these changed values were attenuated in the CA + hypothermia group (Figure 4C,D). In addition, compared with the sham group, caspase 3, a pro-apoptotic protein, was significantly activated in the CA + normothermia group, as revealed by a decrease in pro-caspase 3 (inactive form of caspase 3) and an increase in cleaved caspase 3 (active form of caspase 3) (0.6-fold decrease and 1.7-fold decrease in pro- and cleaved caspase 3, respectively, vs. the sham group). However, the activation of caspase 3 was dramatically attenuated by olanzapine-induced TH (1.8-fold increase and 0.3-fold decrease in pro- and cleaved caspase 3, respectively, in the CA + hypothermia group vs. the sham group) (Figure 4E,F). Collectively, these results indicate that olanzapine-induced TH can effectively attenuate CA-induced apoptotic responses in renal tissues.

### 3.4. Olanzapine-Induced TH Inhibits CA-Induced Oxidative Stress in Renal Tissues

**Figure 5:**
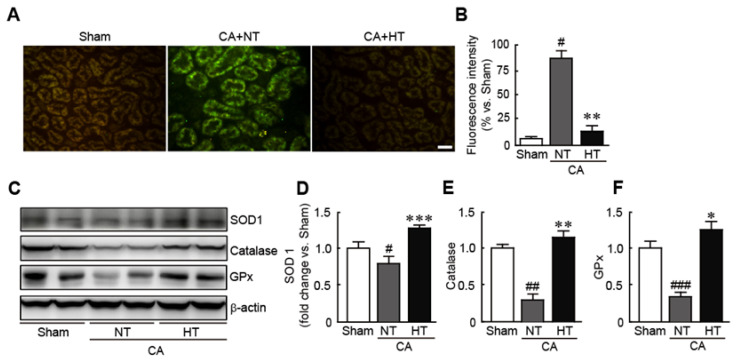
Olanzapine-induced TH inhibits CA-induced oxidative stress in renal tissues. Reactive oxygen species production stained with DCFH-DA (**A**) and fluorescence intensity (**B**) in renal tissues after CA and ROSC with or without olanzapine-induced TH. (**C**–**F**) Western blot analysis for measuring the expression levels of SOD1, catalase, GPx, and *β*-actin proteins (*n* = 3 per group). Significance is represented as # *p* < 0.05, ## *p* < 0.01, and ### *p* < 0.001 vs. the sham group; * *p* < 0.05, ** *p* < 0.01, and *** *p* < 0.001 vs. the CA + NT group. CA, cardiac arrest; TH, therapeutic hypothermia; NT, normothermia; HT, hypothermia; GPx, glutathione peroxidase. Scale bar, 100 µm.

To determine whether hypothermia treatment attenuates oxidative stress in CA-induced rats, ROS production in the renal tissues was assessed by staining with DCFH-DA, an ROS detection dye. The results revealed that the fluorescence intensity of DCFDF-DA, as indicated by green fluorescence, was higher in CA + normothermia group than in the sham group, indicating that CA causes the rapid generation of ROS (81.2% increase in CA + normothermia group vs. the sham group) (Figure 5A,B). In contrast, the fluorescence intensity of DCFH-DA was not detectable in the CA + hypothermia group (6.4% increase in the CA + hypothermia group vs. the sham group) (Figure 5A,B). Moreover, olanzapine-induced TH significantly increased the expression levels of several antioxidants, including SOD1, catalase, and GPx—in contrast to the decreases in the expression levels of these proteins in the CA + normothermia group (0.8, 0.3 and 0.4-fold decreases in SOD1, catalase, and GPx levels, respectively, in the CA + normothermia group and 1.4, 1.2 and 1.3-fold increases, respectively, in the CA + hypothermia group vs. the sham group) (Figure 5C,D). These findings indicate that olanzapine-induced TH can effectively attenuate oxidative stress in CA-induced renal tissues. (Figure 5C,D).

### 3.5. Olanzapine-Induced TH Preserves Mitochondrial Integrity Affected by CA in Renal Tissues

**Figure 6:**
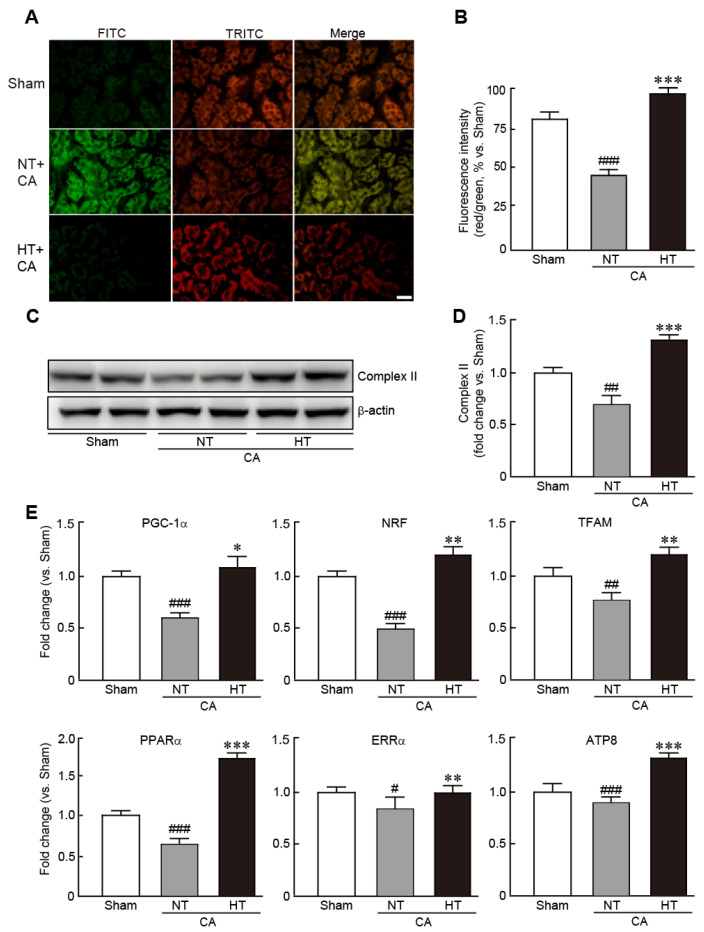
Olanzapine-induced TH preserves mitochondrial integrity affected by CA in renal tissues. Representative photographs (**A**) and intensities of red and green fluorescence (**B**), as observed by JC-1 staining of rat tissues after CA and ROSC with or without olanzapine-induced TH. (**C**,**D**) Western blot analysis for measuring the expression levels of mitochondrial complex II and β-actin proteins. (**E**) qRT-PCR analysis for measuring the mRNA expression levels of mitochondrial biogenesis-related genes (*n* = 3 per group). Significance is represented as # *p* < 0.05, ## *p* < 0.01 and ### *p* < 0.001 vs. the sham group; * *p* < 0.05, ** *p* < 0.01, and *** *p* < 0.001 vs. the CA + NT group. CA, cardiac arrest; TH, therapeutic hypothermia; NT, normothermia; HT, hypothermia. Scale bar, 100 µm.

To determine whether hypothermia could rescue mitochondrial function caused by CA, the levels of MMP, an indicator of mitochondrial function, were measured by JC-1 staining assay in renal tissues from the CA + normothermia and CA + hypothermia groups. CA-induced renal tissues exhibited a collapse of MMP, as indicated by predominant green fluorescence, in comparison with the red florescence noted in the sham group (32.3% decrease in red/green fluorescence intensity vs. the sham group) (Figure 6A,B). However, in the CA + hypothermia group, red fluorescence was remarkably visible, indicating that olanzapine-induced TH effectively preserved MMP (19.0% increase in red/green fluorescence intensity vs. the sham group) (Figure 6A,B). In addition, olanzapine-induced TH preserved the expression of mitochondrial complex II protein, a marker of mitochondrial integrity, whereas this protein was significantly decreased in the CA + hypothermia group (0.7-fold decrease in the CA + normothermia group and 1.3-fold increase in the CA + hypothermia group vs. the sham group) (Figure 6C,D). Similarly, qRT-PCR revealed that the mRNA levels of several mitochondrial biogenesis-related genes, including *PGC1-α*, *NRF*, *TFAM*, *PPARα*, *ERRα* and *ATP8*, were significantly reduced in the CA + normothermia group. However, olanzapine-induced TH preserved the mRNA levels of all these genes (Figure 6E). Collectively, these results demonstrate that olanzapine-induced TH can efficiently preserve the mitochondrial structure and functions in CA-induced renal tissues.

### 3.6. Olanzapine-Induced TH Inhibits CA-Induced ER Stress in Renal Tissues

**Figure 7:**
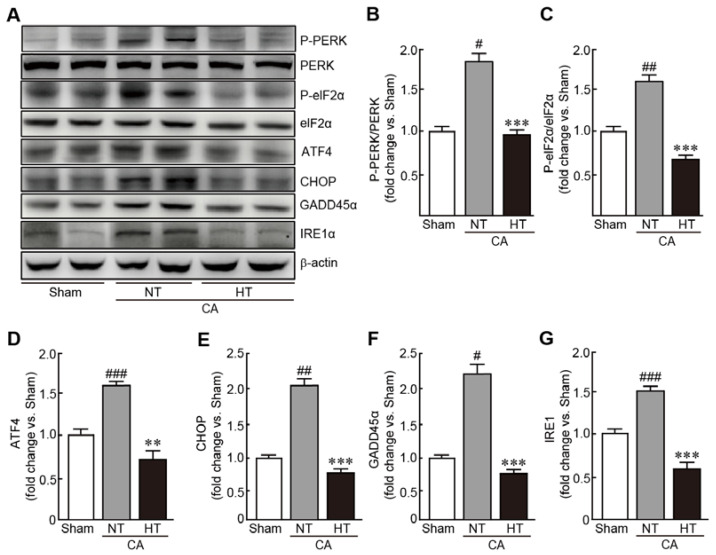
Olanzapine-induced TH inhibits CA-induced ER stress in renal tissues. (**A**–**G**) Western blot analysis for measuring the expression levels of ER stress signaling-related proteins in rat tissues after CA and ROSC with or without olanzapine-induced TH (*n* = 3 per group). Significance is represented as # *p* < 0.05, ## *p* < 0.01, and ### *p* < 0.001 vs. the sham group; ** *p* < 0.01 and *** *p* < 0.001 vs. the CA + NT group. CA, cardiac arrest; TH, therapeutic hypothermia; NT, normothermia; HT, hypothermia.

To evaluate the preventive effects of hypothermia against CA-induced ER stress, expression of several ER stress-related proteins were determined, including PERK, eIF2α, ATF4, CHOP, GADD45α and IRE1α. In the CA + normothermia group, the expression of p-PERK and p-eIF2α were elevated, indicating that these proteins were activated because of CA and ROSC (1.8- and 1.6-fold increases in p-PERK and p-eIF2α, respectively, in the CA-induced group vs. the sham group) (Figure 7A–C). The expression levels of ATF4, CHOP, GADD45α and IRE1α were also elevated in the CA-induced group (1.6, 2.1, 2.2, and 1.2-fold increases in ATF4, CHOP, GADD45α and IRE1α, respectively, in the CA + normothermia group vs. the sham group) (Figure 7A,D–G). Notably, olanzapine-induced TH significantly inhibited the increase in the expression levels of these proteins, keeping their expression levels similar to those in the sham group. These results indicate that olanzapine-induced TH can effectively attenuate ER stress caused by CA and ROSC.

### 3.7. Olanzapine-Induced TH Activates the Sirt3-Related Signaling Pathway in CA-Induced Renal Tissues

**Figure 8:**
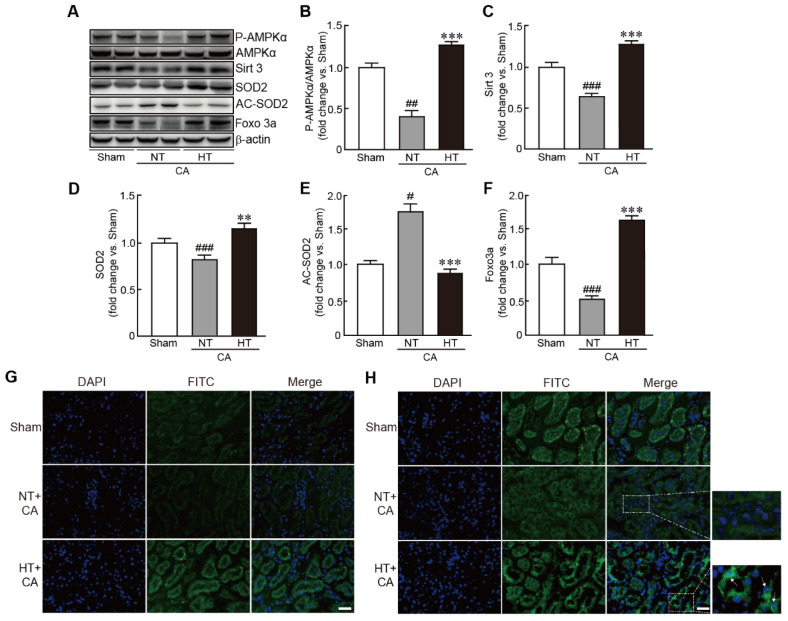
Olanzapine-induced TH activates the Sirt3-related signaling pathway in CA-induced renal tissues. (**A**–**F**) Western blot analysis for measuring the expression levels of proteins involved in the Sirt3 signaling pathway in rat tissues after CA and ROSC with or without olanzapine-induced TH (*n* = 3 per group). Representative photographs of Sirt3 (**G**) and FOXO3a (**H**) protein staining results in rat tissues after CA and ROSC with or without olanzapine-induced TH. Arrows indicate the cells that contain Sirt3 in the nucleus. Significance is represented as ## *p* < 0.01 and ### *p* < 0.001 vs. the sham group; ** *p* < 0.01 and *** *p* < 0.001 vs. the CA + NT group. CA, cardiac arrest; TH, therapeutic hypothermia; NT, normothermia; HT, hypothermia. Scale bar, 100 µm.

To further elucidate the signaling pathway involved in the preventive effect of olanzapine-induced TH against CA, Sirt3, a mitochondrial deacetylase enzyme, and its related signaling pathway were assessed in the CA + hypothermia group. The expression of the p-AMPKα protein was significantly increased in the CA + hypothermia group, indicating the activation of AMPKα; however, the phosphorylation of this protein was significantly decreased in the CA + normothermia group (0.4-fold decrease in the CA + normothermia group and 1.3-fold increase in the CA + hypothermia group vs. the sham group) (Figure 7A,B). Similarly, the expression level of Sirt3 protein was significantly decreased in the CA + normothermia group. However, olanzapine-induced TH suppressed the decrease in the expression level of this protein caused by CA and ROSC (0.7-fold decrease in the CA + normothermia group and 1.3-fold increase in the CA + hypothermia group vs. the sham group) (Figure 7A,C). The expression level of SOD2, a Sirt3 downstream protein, was significantly reduced in the CA + normothermia group. However, olanzapine-induced TH blocked this decrease in expression caused by CA and ROSC, keeping its expression level similar to that in the sham group (0.8-fold decrease and 1.2-fold increase in the CA + normothermia and CA + hypothermia groups, respectively, vs. the sham group) (Figure 8A,D). Immunostaining analysis of Sirt3 protein also revealed that the fluorescence against the protein decreased in CA and ROSC-induced renal cells, whereas olanzapine-induced TH dramatically increased this fluorescence (Figure 7E). The acetylation status of SOD2 protein was found to be increased in the CA + normothermia group (1.8-fold increase in the CA + normothermia group vs. the sham group). Notably, this hyperacetylation of SOD2 caused by CA and ROSC was dramatically blocked on olanzapine-induced TH (0.9-fold decrease in the CA + hypothermia group vs. the sham group) (Figure 8A,E). Finally, the expression level of Foxo3a protein was restored by olanzapine-induced TH against CA and ROSC, as revealed by the results of both Western blot and immunostaining analyses. In particular, some cells were found to contain Foxo3a protein within the nucleus (Figure 8A,H). Collectively, these findings indicate that the Sirt3-related signaling pathway mediates the preventive effect of olanzapine-induced TH against CA and ROSC.

## 4. Discussion

Olanzapine, an atypical antipsychotic agent, is used to treat psychotic illnesses, such as schizophrenia and bipolar I disorder [29,30], mediated by the antagonism of 5-HT_2A/C_ and dopamine receptors [31]. Many clinical studies have reported that olanzapine also induces hypothermia [32,33]. Furthermore, in vivo studies using experimental animals have established the hypothermia-inducing effect of olanzapine. In one study, olanzapine remarkably induced a hypothermic response in rats after daily administration for 14 days [34]. In another study, olanzapine caused the rapid onset of hypothermia in rats, with the maximal effect being noted 2 h after administration [35]. Therefore, in the present study, we used olanzapine to induce hypothermia after the induction of CA. We found that olanzapine dramatically induced hypothermia, as revealed by a rapid decrease in body temperature within 5 min after the injection of olanzapine. Furthermore, olanzapine-induced TH dramatically increased the survival rate of rats after CA and ROSC. 

Renal dysfunction can occur in CA patients after CPR [36]. The restoration of blood flow due to ROSC after CA may cause renal injury, known as I/R injury [10]. Consequently, renal I/R injury can cause AKI, further contributing to the incidence of many clinical conditions and high mortality [37]. Therefore, in the present study, we focused on elucidating the preventive effect of TH against renal injury induced by ROSC after CA. For this purpose, we established a CA/resuscitation-induced rat model and applied TH to this model using olanzapine after ROSC. We found that TH induced by olanzapine administration for 6 h effectively inhibited the pathohistological alterations in renal tissues in resuscitated rats after CA—e.g., the necrosis and erosion of brush borders in renal tubules and dilation of renal glomerular capillaries. In addition, the serum levels of renal injury markers, such as BUN and creatinine, were suppressed by olanzapine-induced TH in rats after CA and ROSC. Furthermore, the number of cells undergoing apoptotic cell death was reduced and the expression of apoptosis-related proteins was blocked by olanzapine-induced TH, indicating that apoptotic cell death in renal cells after CA and ROSC was attenuated by olanzapine-induced TH. 

Oxidative stress is the important factor inducing AKI following I/R injury [38]. Much evidence has emerged that after the overproduction of ROS, the balance between pro-oxidants and antioxidants is collapsed, and subsequently, oxidative stress is provoked in renal tissues upon renal ischemia, followed by reperfusion [38]. Hence, we determined the protective effects of olanzapine-induced TH on oxidative stress in the kidney of rats after CA and ROSC. We found that olanzapine-induced TH effectively ameliorated ROS overproduction and preserved the expression levels of several antioxidants, including SOD1, catalase, and GPx. 

The mitochondria play a significant role in the incidence of oxidative stress. The mitochondria are believed to be a major source of ROS, which are produced during ATP production by the electron transport chain [39]. In addition, the mitochondria possess potent antioxidant systems consisting of SOD, catalase, and GPx that protect the mitochondria themselves from ROS-mediated oxidative damage [40]. Therefore, under normal mitochondrial conditions, the balance between pro- and antioxidant systems can be maintained. Indeed, mitochondrial dysfunctions, which are closely associated with renal I/R injury, cause ROS accumulation, in turn leading to oxidative stress [40,41]. In this regard, we detected the presence of mitochondrial dysfunction by assessing the levels of MMP, a mitochondrial structural protein, and several biogenesis-related genes. Notably, olanzapine-induced TH effectively prevented mitochondrial dysfunction induced by CA and ROSC. 

ER is a subcellular organelle responsible for the folding and post-translational maturation of membrane-bound and secreted proteins [42]. As this function of ER is sensitive to various external stimuli, unfolded and misfolded proteins get accumulated inside the ER lumen. This phenomenon is known as ER stress and is followed by activating of the unfolded protein response (UPR) to overcome this protein-folding issue. UPR could contribute to oxidative stress [43]. ER stress can cause mitochondrial dysfunction and subsequent mitochondrial ROS accumulation, resulting in oxidative stress [44]. Therefore, we determined the preventive effects of TH against ER stress responses by determining the expression levels of ER-stress related proteins in rats after CA and ROSC. We found that CA and ROSC activated or increased the expression levels of these proteins; however, these effects were dramatically attenuated by olanzapine-induced TH. Therefore, our results indicate that olanzapine-induced TH can effectively rescue the renal tissues from oxidative damage by inhibiting mitochondrial dysfunction and ER stress.

For understanding the mechanism underlying the preventive effects of olanzapine-induced TH, we focused on the Sirt3-related signaling pathway. Sirt3 is an NAD^+^-dependent deacetylase that is mainly present in the mitochondria [45]. It has various biological functions, such as mitochondrial homeostasis, energy metabolism, lipid metabolism, and longevity [46]. In particular, Sirt3 is involved in the defense system against oxidative stress by regulating the activities of various substrates, such as AMPK, SOD2, and FOXO3a [47]. In addition, Sirt3 protects against AKI, as proven by gain- or loss-of-function research targeting Sirt3 [48,49]. In accordance with these findings, we found that olanzapine-induced TH preserved the expression of Sirt3 protein in rats after CA and ROSC. Furthermore, the expression of AMPK and SOD2 proteins, which are downstream targets of Sirt3, was preserved by olanzapine-induced TH. The acetylation of SOD2 was reduced, which may be attributed to the activation of Sirt3 due to olanzapine-induced TH. As acetylation is considered to inhibit its enzymatic activity, SOD2 protein is activated by olanzapine-induced TH. FOXO3a is a transcription factor with the conserved forkhead DNA-binding domain which has a critical role for regulation of gene transcription [50]. In particular, FOXO3a, another target of Sirt3, plays a pivotal role in preventing oxidative stress by regulating the transcription of several genes involved in oxidative stress-related signaling pathways [51]. We found that olanzapine-induced TH increased the expression of FOXO3a and that FOXO3a was present in the nuclei of some cells, indicating that FOXO3a is activated as a result of olanzapine-induced TH after CA and ROSC.

## 5. Conclusions

The present study revealed that olanzapine-induced TH alleviates AKI after CA and ROSC. Furthermore, the preventive effects of olanzapine-induced TH are mediated by the inhibition of mitochondrial dysfunction through the activation of the Sirt3-related signaling pathway. Therefore, we propose that olanzapine-induced TH may be used for the treatment of CA followed by ROSC. Furthermore, olanzapine-induced TH may be applied to the procedures for organ donors after cardiac arrest and patients in the ICU who survive CA, to improve resuscitation.

## Figures and Tables

**Table 1:** Primer sequences.

Genes	Accesion No.	Primers	
*PGC-1α*	NM_031347.1	forward	5′-ACCGTAAATCTGCGGGATGA-3′
		reverse	5′-AGTTTCATTCGACCTGCGTAAAGTA-3′
*NRF-1*	NM_001100708.1	forward	5′-CACTCTGGCTGAAGCCACCTTAC-3′
		reverse	5′-TCACGGCTTTGCTGATGGTC-3′
*TFAM*	NM_031326.1	forward	5′-TGAAGCTTGTAAATCAGGCTTGGA-3′
		reverse	5′-GAGATCACTTCGCCCAACTTCAG-3′
*PPARα*	NM_013196.1	forward	5′-GGCAATGCACTGAACATCGAG-3′
		reverse	5′-GCCGAATAGTTCGCCGAAAG-3′
*ERRα*	NM_001008511.2	forward	5′-GCTGAAAGCTCTGGCCCTTG-3′
		reverse	5′-TGCTCCACAGCCTCAGCAT-3′
*ATP8*	NC_012920.1	forward	5′-TGCCACAACTAGACACATCCA-3′
		reverse	5′-TGTGGGGGTAATGAAAGAGG-3′
*18S rRNA* (internal control)	NM_213557.1	forward	5′-AAGTTTCAGCACATCCTGCGAGTA-3′
		reverse	5′-TTGGTGAGGTCAATGTCTGCTTTC-3′

## Data Availability

Data is contain within the article.
